# Partial Segmental Thrombosis of the Corpus Cavernosum (PSTCC) diagnosed by contrast-enhanced ultrasound: a case report

**DOI:** 10.1186/1471-2490-14-100

**Published:** 2014-12-17

**Authors:** Stephanie Sauer, Jan P Goltz, Tobias Gassenmaier, Andreas S Kunz, Thorsten A Bley, Detlef Klein, Bernhard Petritsch

**Affiliations:** University Hospital Würzburg, Institute of Diagnostic and Interventional Radiology, Oberdürrbacher Str. 6, 97080 Würzburg, Germany; University Hospital Schleswig-Holstein, Campus Lübeck, Clinic for Radiology and Nuclear Medicine, Ratzeburger Allee 160, 23538 Lübeck, Germany

**Keywords:** Penile thrombosis, Corpus cavernosum, Priapism, Contrast-enhanced ultrasound, MRI

## Abstract

**Background:**

Partial segmental thrombosis of the corpus cavernosum (PSTCC) is a rare disease predominantly occurring in young men. Cardinal symptoms are pain and perineal swelling. Although several risk factors are described in the literature, the exact etiology of penile thrombosis remains unclear in most cases. MRI or ultrasound (US) is usually used for diagnosing this condition.

**Case presentation:**

We report a case of penile thrombosis after left-sided varicocele ligature in a young patient. The diagnosis was established using contrast-enhanced ultrasound (CEUS) and was confirmed by contrast-enhanced magnetic resonance imaging (ceMRI). Successful conservative treatment consisted of systemic anticoagulation using low molecular weight heparin and acetylsalicylic acid.

**Conclusion:**

PSTCC is a rare condition in young men and appears with massive pain and perineal swelling. In case of suspected PSTCC utilization of CEUS may be of diagnostic benefit.

## Background

Partial segmental thrombosis of the corpus cavernosum (PSTCC) is a rare disease predominantly occurring in young men [1]. Priapism (prolonged and painful penile erection) must be discerned from actual thrombosis of the corpus cavernosum [2]. PSTCC is characterized by thrombosis that most often is unilateral andlocated in the proximal part of the corpus cavernosum. Cardinal symptoms are massive pain and perineal swelling [3]. The etiology of penile thrombosis remains unclear. Several risk factors are described in the literature [2–6]. In most patients an excellent outcome is achieved with conservative treatment [7, 8].

MRI or ultrasound (US) are usually used for diagnosis [9]. This report describes the use of contrast-enhanced ultrasound (CEUS) for assessment of penile thrombosis.

### Case presentation

A 23-year-old male was admitted to our university hospital with severe pain and a history of progressive perineal swelling over a period of two weeks. His medical history was non-contributory except a left-sided varicocele ligature performed six years ago. No sexual trauma, dysuria or urethral discharge was reported by the patient. Excessive bicycle riding, masturbation, marijuana or cocaine consumption as well as recent long distance flights were negated.

Physical examination confirmed the absence of priapism. A firm, fixed and extremely painful mass was palpable at the proximal penis. The complete blood count, electrolytes, coagulation profile, CRP and urine analysis were unremarkable.

**Figure 1:**
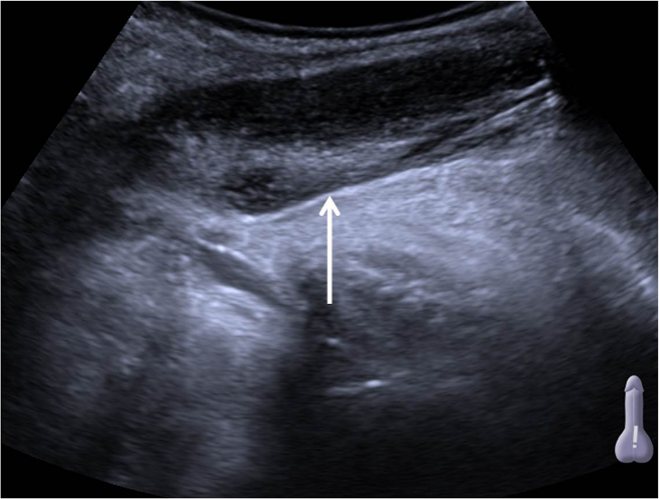
**Ultrasound scan (longitudinal axis) of the right corpus cavernosum.** Arrow shows thrombus in the proximal part.

**Figure 2:**
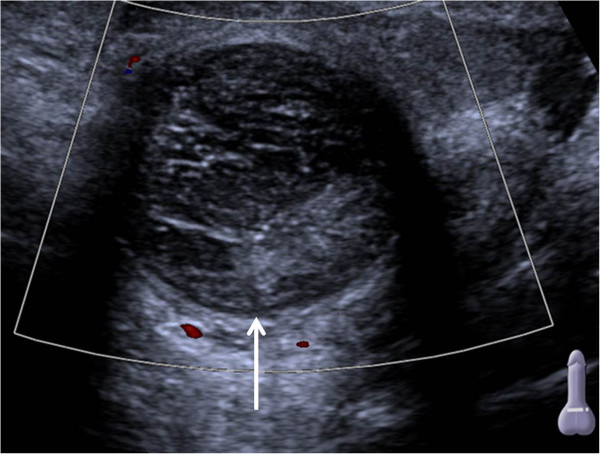
**Color Doppler ultrasound (transversal axis) of the proximal penis.** Arrow shows absence of flow in the thrombus.

Gray-scaled ultrasound (Siemens Acuson S2000, Siemens Sector Healthcare, Erlangen, Germany) combined with color Doppler of the penis using a 6 MHz transducer (Siemens 6C1 HD, Siemens Sector Healthcare, Erlangen, Germany) revealed an elongated hypoechogenic mass (Figure 1, arrow) with irregular hyperechogenic internal echoes. Color Doppler ultrasound demonstrated absence of flow within the inhomogeneous mass (Figure 2, arrow).

**Figure 3:**
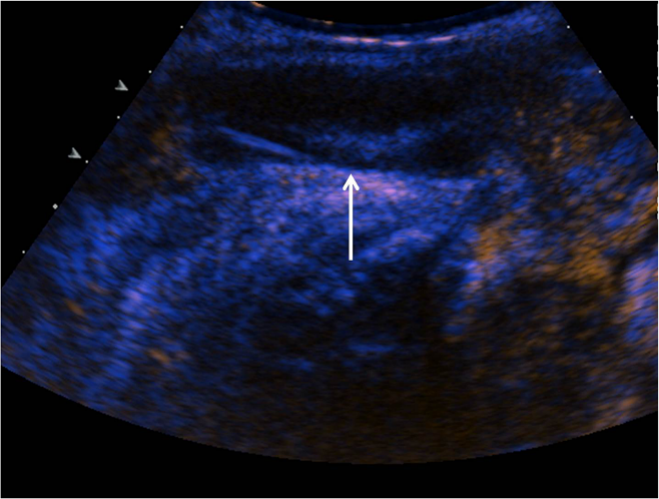
**Contrast-enhanced ultrasound shows lack of perfusion and confirms the diagnosis of PSTCC (arrow).**

Contrast-enhanced ultrasound (Acuson Sequoia with 4 MHz transducer, Siemens Sector Healthcare, Erlangen, Germany; 1.2 ml SonoVue®, Bracco Imaging, Milano, Italy) revealed a lack of contrast enhancement in the hypoechogenic mass. This was interpreted as segmental thrombosis of the corpus cavernosum (Figure 3, arrow).

**Figure 4:**
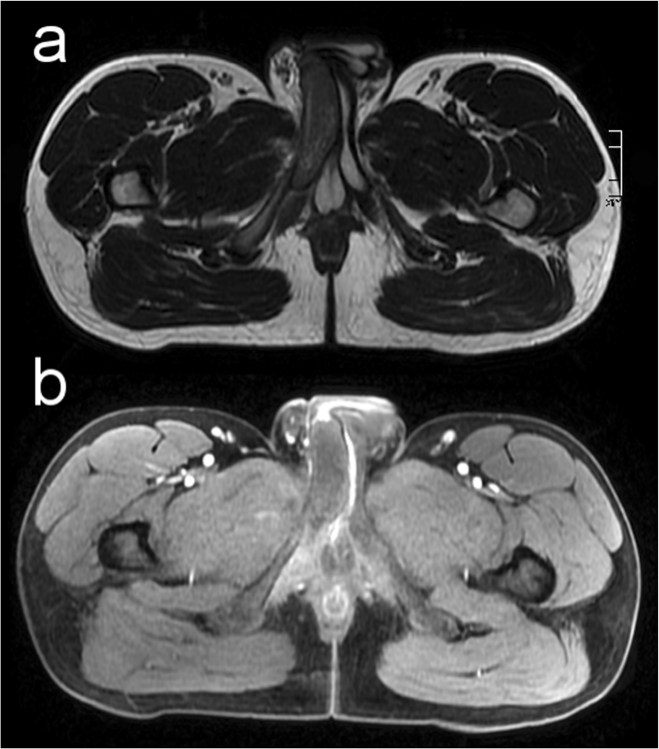
**Transversal T2-weighted (a) and fat saturated T1-weighted (b) MRI images through the basis of the penis.** Arrows show thrombosis of the right proximal corpus cavernosum.

For confirmation of this diagnosis a MRI study (1.5 T Siemens Avanto, Siemens Sector Healthcare, Erlangen, Germany) of the pelvis was performed. Transversal T2-weighted images of the pelvis showed a hypointense thrombus of the right proximal corpus cavernosum (Figure 4a, white arrow). T1-weighted fat saturated transversal scans after intravenous contrast medium injection (Gadovist®, Bayer HealthCare AG, Leverkusen, Germany) revealed an intermediate signal and a lack of contrast uptake in the right proximal corpus cavernosum (Figure 4b, black arrow) consistent with sub-acute partial segmental thrombosis of the corpus cavernosum.

Conservative treatment was initiated and consisted of anticoagulation with low molecular weight heparin (Enoxaparin 40 mg once daily) and acetylsalicylic acid (100 mg once daily).

## Discussion

Partial segmental thrombosis of the corpus cavernosum (PSTCC) is a rare urological disease which primarily occurs in young men with a mean age of 30 years (range 18–59 years) [1]. Differential diagnoses include inflammatory processes as well as solid tumors [10].

PSTCC´s etiology is poorly understood. Although several risk factors have been described, the exact etiology still remains unclear. Recently Ilicki et al. [1] postulated a two-hit model in which first a transverse membrane, dividing the corpus into a proximal and distal portion, and second a triggering event (microtrauma) is required for the emergence of corpus cavernosum thrombosis. In the presented case no solid membrane as a potentially flow-limiting factor was identified, neither in CEUS nor in MRI. Such membranes may predestinate the emergence of PSTCC but their presence may not be mandatory.

Risk factors for development of PSTCC include haematological abnormalities (sickle cell disease, leukemia, homocysteinemia), abuse of alcohol or drugs (marijuana, cocaine), hypercoagulability associated with malignancy and long distance flights as well as microtrauma after coitus or extended cycling [1–6].

### Imaging

Standard imaging modalities include color coded duplex sonography, MRI and CT imaging. The benefits of contrast enhanced MRI have been demonstrated by several authors [1, 2, 6, 11]. In recent years, CEUS has been found to be a cost- efficient alternative in diagnosis of organ and soft tissue pathologies, especially in patients with contraindications for MRI.

This case report outlines the utility of CEUS as an additional cost-saving diagnostic tool for assessment of PSTCC, especially in patients with contraindications for MRI.

### Treatment

The first cases of PSTCC were described in 1976 [12]. At that time surgical treatment was the method of choice. Nowadays, as in this case, conservative treatment with systemic anticoagulation appears to be an excellent and reliable therapeutic option. Recent literature shows that all patients treated conservatively have maintained erectile function [5, 7, 8, 13]. Surgery is reserved for patients in whom conservative management fails [5].

## Conclusions

PSTCC is a rare condition that predominantly occurs in young men and is accompanied with massive pain and perineal swelling. There are several risk factors described, including abuse of alcohol or drugs, hypercoagulability associated with malignancy and long distance flights as well as microtrauma after coitus or extended bicycle riding. CEUS may be of diagnostic benefit if one suspects PSTCC. Larger studies are warranted to verify this hypothesis. Conservative treatment with systemic anticoagulation has shown excellent results concerning pain relief and erectile dysfunction.

## Consent

Written informed consent was obtained from the patient for publication of this case report and any accompanying images. A copy of the written consent is available for review by the Editor of this journal.

## Abbreviations

PSTCC: Partial segmental thrombosis of the corpus cavernosum
ceMRI: Contrast-enhanced magnetic resonance imaging
CEUS: Contrast-enhanced ultrasound.
